# *N*′-(2,3-Dimethoxy­benzyl­idene)-2,4-dihydroxy­benzohydrazide

**DOI:** 10.1107/S1600536810012134

**Published:** 2010-04-10

**Authors:** You-Yue Han, Qiu-Rong Zhao

**Affiliations:** a Department of Chemistry and Life Science, Chuzhou University, Chuzhou, Anhui 239000, People’s Republic of China

## Abstract

In the title compound, C_16_H_16_N_2_O_5_, the dihedral angle between the two benzene rings is 8.5 (3)° and the mol­ecule adopts an *E* configuration with respect to the C=N bond. There is an intra­molecular N—H⋯O hydrogen bond in the mol­ecule, which generates an *S*(6) ring. In the crystal, mol­ecules are linked through inter­molecular O—H⋯O hydrogen bonds, forming layers parallel to the *bc* plane.

## Related literature

For related structures and background information, see: Han & Zhao (2010*a* ▶,*b* ▶). For reference structural data, see: Allen *et al.* (1987 ▶).
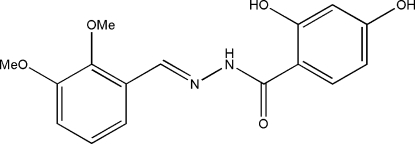


## Experimental

### 

#### Crystal data

C_16_H_16_N_2_O_5_
*M* *_r_* = 316.31
Monoclinic, 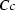

*a* = 24.918 (4) Å
*b* = 5.0291 (8) Å
*c* = 13.075 (2) Å
β = 118.994 (2)°
*V* = 1433.1 (4) Å^3^
*Z* = 4
Mo *K*α radiation
μ = 0.11 mm^−1^
*T* = 298 K
0.20 × 0.20 × 0.18 mm

#### Data collection

Bruker SMART CCD diffractometer
Absorption correction: multi-scan (*SADABS*; Bruker, 2001 ▶) *T*_min_ = 0.978, *T*_max_ = 0.980
3934 measured reflections
1549 independent reflections
1247 reflections with *I* > 2σ(*I*)
*R*_int_ = 0.047

#### Refinement

*R*[*F* ^2^ > 2σ(*F* ^2^)] = 0.034
*wR*(*F* ^2^) = 0.089
*S* = 0.78
1549 reflections
215 parameters
3 restraints
H atoms treated by a mixture of independent and constrained refinement
Δρ_max_ = 0.15 e Å^−3^
Δρ_min_ = −0.15 e Å^−3^

### 

Data collection: *SMART* (Bruker, 2007 ▶); cell refinement: *SAINT* (Bruker, 2007 ▶); data reduction: *SAINT*; program(s) used to solve structure: *SHELXTL* (Sheldrick, 2008 ▶); program(s) used to refine structure: *SHELXTL*; molecular graphics: *SHELXTL*; software used to prepare material for publication: *SHELXTL*.

## Supplementary Material

Crystal structure: contains datablocks global, I. DOI: 10.1107/S1600536810012134/hb5385sup1.cif

Structure factors: contains datablocks I. DOI: 10.1107/S1600536810012134/hb5385Isup2.hkl

Additional supplementary materials:  crystallographic information; 3D view; checkCIF report

## Figures and Tables

**Table 1:** Hydrogen-bond geometry (Å, °)

*D*—H⋯*A*	*D*—H	H⋯*A*	*D*⋯*A*	*D*—H⋯*A*
N1—H1*A*⋯O1	0.89 (1)	1.93 (3)	2.661 (3)	138 (4)
O1—H1⋯O3^i^	0.82	2.16	2.872 (3)	146
O2—H2⋯O3^ii^	0.82	1.90	2.706 (3)	166

Symmetry codes: (i) 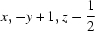; (ii) 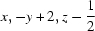.

**Fig. 1.**
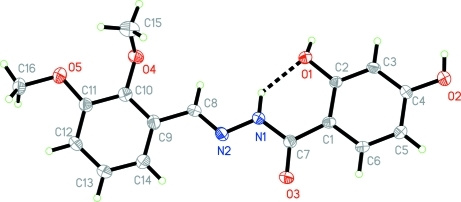
The molecular structure of (I) with 30% probability displacement ellipsoids for non-H atoms. Intramolecular N–H···O hydrogen bond is shown as a dashed line.

**Fig. 2.**
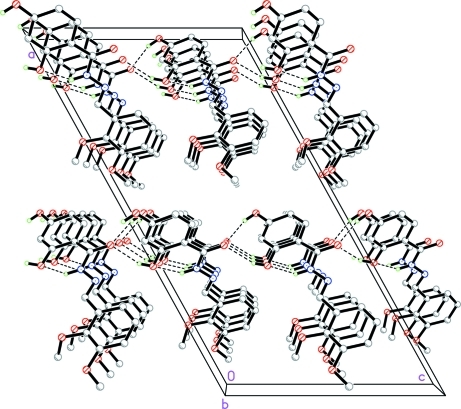
The molecular packing of (I), viewed along the b axis. Hydrogen bonds are shown as dashed lines.

## supplementary crystallographic information

### Comment

As part of our ongoing structural studies of hydrazone compounds (Han & Zhao, 2010a,b), we now report the sturcture of the title compound, (I).

In the molecule of the title compound, Fig. 1, the dihedral angle between the two benzene rings is 8.5 (3)°. The molecule adopts an *E* configuration with respect to the C═N bond. There is an intramolecular N–H···O hydrogen bond (Table 1) in the molecule.

In the crystal structure, molecules are linked through intermolecular O–H···O hydrogen bonds (Table 1) to form layers parallel to the *bc* plane (Fig. 2).

### Experimental

A mixture of 2,3-dimethoxybenzaldehyde (0.166 g, 1 mmol) and 2,4-dihydroxybenzohydrazide (0.168 g, 1 mmol) in 50 ml me thanol was stirred at room temperature for 1 h. The mixture was filtered to remove impurities, and then left at room temperature. After a few days, colourless blocks of (I) were formed.

### Refinement

H1A was located in a difference Fourier map and refined isotropically, with the N–H distance restrained to 0.90 (1) Å. The other H atoms were positioned geometrically and refined using the riding-model approximation, with C–H = 0.93 or 0.96 Å, and O–H = 0.82 Å, and U_iso_(H) = 1.2U_eq_(C) or U_iso_(H) = 1.5U_eq_(methyl C and O). In the absence of significant anomalous dispersion effects, Friedel pairs were averaged.

### Crystal data

**Table:**

C_16_H_16_N_2_O_5_	*F*(000) = 664
*M**_r_* = 316.31	*D*_x_ = 1.466 Mg m^−^^3^
Monoclinic, *C**c*	Mo *K*α radiation, λ = 0.71073 Å
Hall symbol: C -2yc	Cell parameters from 1397 reflections
*a* = 24.918 (4) Å	θ = 2.7–25.0°
*b* = 5.0291 (8) Å	µ = 0.11 mm^−^^1^
*c* = 13.075 (2) Å	*T* = 298 K
β = 118.994 (2)°	Block, colourless
*V* = 1433.1 (4) Å^3^	0.20 × 0.20 × 0.18 mm
*Z* = 4	

### Data collection

**Table:**

Bruker SMART CCD diffractometer	1549 independent reflections
Radiation source: fine-focus sealed tube	1247 reflections with *I* > 2σ(*I*)
graphite	*R*_int_ = 0.047
ω scans	θ_max_ = 27.0°, θ_min_ = 1.9°
Absorption correction: multi-scan (*SADABS*; Bruker, 2001)	*h* = −23→31
*T*_min_ = 0.978, *T*_max_ = 0.980	*k* = −6→6
3934 measured reflections	*l* = −16→16

### Refinement

**Table:**

Refinement on *F*^2^	Primary atom site location: structure-invariant direct methods
Least-squares matrix: full	Secondary atom site location: difference Fourier map
*R*[*F*^2^ > 2σ(*F*^2^)] = 0.034	Hydrogen site location: inferred from neighbouring sites
*wR*(*F*^2^) = 0.089	H atoms treated by a mixture of independent and constrained refinement
*S* = 0.78	*w* = 1/[σ^2^(*F*_o_^2^) + (0.0677*P*)^2^ + 0.0824*P*] where *P* = (*F*_o_^2^ + 2*F*_c_^2^)/3
1549 reflections	(Δ/σ)_max_ = 0.001
215 parameters	Δρ_max_ = 0.15 e Å^−^^3^
3 restraints	Δρ_min_ = −0.15 e Å^−^^3^

### Special details

**Table:**

Geometry. All esds (except the esd in the dihedral angle between two l.s. planes) are estimated using the full covariance matrix. The cell esds are taken into account individually in the estimation of esds in distances, angles and torsion angles; correlations between esds in cell parameters are only used when they are defined by crystal symmetry. An approximate (isotropic) treatment of cell esds is used for estimating esds involving l.s. planes.
Refinement. Refinement of *F*^2^ against ALL reflections. The weighted *R*-factor wR and goodness of fit *S* are based on *F*^2^, conventional *R*-factors *R* are based on *F*, with *F* set to zero for negative *F*^2^. The threshold expression of *F*^2^ > σ(*F*^2^) is used only for calculating *R*-factors(gt) etc. and is not relevant to the choice of reflections for refinement. *R*-factors based on *F*^2^ are statistically about twice as large as those based on *F*, and *R*- factors based on ALL data will be even larger.

### Fractional atomic coordinates and isotropic or equivalent isotropic displacement parameters (Å^2^)

**Table:**

	*x*	*y*	*z*	*U*_iso_*/*U*_eq_	
N1	0.34984 (10)	0.3854 (5)	0.19993 (19)	0.0380 (5)	
N2	0.32187 (10)	0.2191 (4)	0.24411 (19)	0.0393 (5)	
O1	0.35804 (9)	0.5925 (4)	0.02075 (15)	0.0441 (5)	
H1	0.3604	0.5886	−0.0396	0.066*	
O2	0.49643 (9)	1.3145 (4)	0.09920 (16)	0.0456 (5)	
H2	0.4745	1.3395	0.0289	0.068*	
O3	0.41233 (10)	0.5503 (4)	0.37839 (17)	0.0460 (5)	
O4	0.18657 (10)	−0.2434 (4)	0.00073 (18)	0.0474 (5)	
O5	0.11485 (10)	−0.5565 (4)	0.05383 (18)	0.0526 (6)	
C1	0.42086 (11)	0.7354 (5)	0.2189 (2)	0.0317 (5)	
C2	0.40165 (11)	0.7604 (5)	0.0990 (2)	0.0310 (5)	
C3	0.42595 (12)	0.9539 (5)	0.0580 (2)	0.0348 (5)	
H3	0.4117	0.9707	−0.0219	0.042*	
C4	0.47143 (12)	1.1230 (5)	0.1358 (2)	0.0339 (6)	
C5	0.49350 (13)	1.0928 (5)	0.2551 (2)	0.0409 (6)	
H5	0.5254	1.1995	0.3082	0.049*	
C6	0.46786 (12)	0.9044 (5)	0.2941 (2)	0.0381 (6)	
H6	0.4825	0.8886	0.3741	0.046*	
C7	0.39455 (12)	0.5504 (5)	0.2719 (2)	0.0331 (6)	
C8	0.28150 (12)	0.0637 (5)	0.1698 (2)	0.0410 (6)	
H8	0.2739	0.0663	0.0929	0.049*	
C9	0.24668 (12)	−0.1189 (6)	0.2024 (2)	0.0397 (6)	
C10	0.19806 (13)	−0.2619 (5)	0.1154 (2)	0.0373 (6)	
C11	0.16267 (13)	−0.4294 (5)	0.1442 (2)	0.0402 (6)	
C12	0.17733 (14)	−0.4550 (6)	0.2600 (3)	0.0494 (7)	
H12	0.1539	−0.5652	0.2800	0.059*	
C13	0.22640 (17)	−0.3188 (7)	0.3461 (3)	0.0568 (8)	
H13	0.2365	−0.3425	0.4239	0.068*	
C14	0.26058 (14)	−0.1489 (6)	0.3189 (3)	0.0511 (7)	
H14	0.2928	−0.0541	0.3777	0.061*	
C15	0.13382 (18)	−0.0901 (7)	−0.0739 (3)	0.0626 (9)	
H15A	0.0978	−0.1745	−0.0802	0.094*	
H15B	0.1305	−0.0775	−0.1500	0.094*	
H15C	0.1377	0.0850	−0.0418	0.094*	
C16	0.07976 (15)	−0.7352 (7)	0.0833 (3)	0.0522 (8)	
H16A	0.1058	−0.8754	0.1318	0.078*	
H16B	0.0471	−0.8098	0.0130	0.078*	
H16C	0.0628	−0.6404	0.1247	0.078*	
H1A	0.3388 (18)	0.390 (8)	0.1239 (12)	0.080*	

### Atomic displacement parameters (Å^2^)

**Table:**

	*U*^11^	*U*^22^	*U*^33^	*U*^12^	*U*^13^	*U*^23^
N1	0.0413 (13)	0.0432 (12)	0.0305 (11)	−0.0079 (10)	0.0183 (10)	0.0016 (10)
N2	0.0402 (13)	0.0420 (12)	0.0389 (12)	−0.0023 (10)	0.0218 (11)	0.0052 (10)
O1	0.0542 (12)	0.0502 (11)	0.0279 (9)	−0.0175 (9)	0.0200 (9)	−0.0075 (8)
O2	0.0498 (13)	0.0441 (10)	0.0430 (11)	−0.0080 (9)	0.0225 (10)	0.0053 (9)
O3	0.0624 (12)	0.0486 (11)	0.0298 (10)	−0.0120 (10)	0.0245 (9)	−0.0017 (8)
O4	0.0543 (11)	0.0553 (11)	0.0400 (10)	−0.0028 (10)	0.0287 (9)	−0.0038 (9)
O5	0.0572 (13)	0.0550 (12)	0.0458 (11)	−0.0207 (10)	0.0250 (10)	−0.0085 (10)
C1	0.0341 (14)	0.0323 (13)	0.0292 (13)	0.0034 (10)	0.0158 (11)	0.0007 (10)
C2	0.0334 (14)	0.0320 (12)	0.0283 (13)	−0.0011 (11)	0.0156 (12)	−0.0025 (10)
C3	0.0403 (14)	0.0374 (13)	0.0271 (12)	0.0005 (11)	0.0167 (11)	0.0003 (10)
C4	0.0369 (13)	0.0315 (13)	0.0364 (14)	−0.0001 (11)	0.0200 (12)	0.0023 (11)
C5	0.0414 (15)	0.0442 (15)	0.0329 (14)	−0.0100 (12)	0.0147 (12)	−0.0065 (11)
C6	0.0422 (14)	0.0434 (14)	0.0256 (12)	−0.0048 (12)	0.0140 (11)	−0.0029 (10)
C7	0.0386 (14)	0.0347 (13)	0.0289 (13)	0.0000 (11)	0.0188 (11)	−0.0017 (10)
C8	0.0422 (16)	0.0453 (15)	0.0337 (14)	−0.0037 (12)	0.0169 (12)	0.0042 (12)
C9	0.0393 (15)	0.0403 (14)	0.0405 (15)	−0.0003 (12)	0.0203 (12)	0.0036 (12)
C10	0.0409 (14)	0.0359 (14)	0.0384 (14)	0.0023 (11)	0.0219 (12)	−0.0012 (11)
C11	0.0421 (15)	0.0377 (14)	0.0445 (16)	−0.0027 (12)	0.0240 (13)	−0.0027 (12)
C12	0.0541 (18)	0.0520 (16)	0.0466 (17)	−0.0134 (14)	0.0281 (15)	0.0027 (14)
C13	0.0626 (19)	0.0684 (19)	0.0375 (16)	−0.0169 (17)	0.0228 (15)	0.0044 (15)
C14	0.0495 (17)	0.0559 (17)	0.0392 (16)	−0.0146 (14)	0.0146 (14)	0.0000 (13)
C15	0.073 (2)	0.070 (2)	0.0394 (17)	0.0109 (18)	0.0230 (16)	0.0041 (15)
C16	0.0493 (18)	0.0521 (17)	0.058 (2)	−0.0127 (14)	0.0284 (16)	−0.0027 (14)

### Geometric parameters (Å, °)

**Table:**

N1—C7	1.342 (3)	C5—C6	1.371 (4)
N1—N2	1.382 (3)	C5—H5	0.9300
N1—H1A	0.893 (10)	C6—H6	0.9300
N2—C8	1.272 (3)	C8—C9	1.461 (4)
O1—C2	1.365 (3)	C8—H8	0.9300
O1—H1	0.8200	C9—C10	1.394 (4)
O2—C4	1.354 (3)	C9—C14	1.397 (4)
O2—H2	0.8200	C10—C11	1.397 (3)
O3—C7	1.239 (3)	C11—C12	1.380 (4)
O4—C10	1.386 (3)	C12—C13	1.378 (4)
O4—C15	1.425 (4)	C12—H12	0.9300
O5—C11	1.364 (3)	C13—C14	1.369 (4)
O5—C16	1.430 (3)	C13—H13	0.9300
C1—C6	1.394 (3)	C14—H14	0.9300
C1—C2	1.406 (3)	C15—H15A	0.9600
C1—C7	1.489 (3)	C15—H15B	0.9600
C2—C3	1.384 (3)	C15—H15C	0.9600
C3—C4	1.387 (4)	C16—H16A	0.9600
C3—H3	0.9300	C16—H16B	0.9600
C4—C5	1.389 (4)	C16—H16C	0.9600
			
C7—N1—N2	119.8 (2)	C9—C8—H8	119.2
C7—N1—H1A	118 (3)	C10—C9—C14	119.5 (2)
N2—N1—H1A	122 (3)	C10—C9—C8	119.3 (2)
C8—N2—N1	115.2 (2)	C14—C9—C8	121.2 (3)
C2—O1—H1	109.5	O4—C10—C9	119.4 (2)
C4—O2—H2	109.5	O4—C10—C11	120.4 (2)
C10—O4—C15	114.5 (2)	C9—C10—C11	120.2 (2)
C11—O5—C16	116.9 (2)	O5—C11—C12	124.2 (2)
C6—C1—C2	116.6 (2)	O5—C11—C10	116.7 (2)
C6—C1—C7	117.5 (2)	C12—C11—C10	119.1 (2)
C2—C1—C7	125.9 (2)	C11—C12—C13	120.5 (3)
O1—C2—C3	118.9 (2)	C11—C12—H12	119.7
O1—C2—C1	119.8 (2)	C13—C12—H12	119.7
C3—C2—C1	121.2 (2)	C14—C13—C12	121.1 (3)
C4—C3—C2	120.2 (2)	C14—C13—H13	119.5
C4—C3—H3	119.9	C12—C13—H13	119.5
C2—C3—H3	119.9	C13—C14—C9	119.6 (3)
O2—C4—C3	122.0 (2)	C13—C14—H14	120.2
O2—C4—C5	118.4 (2)	C9—C14—H14	120.2
C3—C4—C5	119.6 (2)	O4—C15—H15A	109.5
C6—C5—C4	119.4 (2)	O4—C15—H15B	109.5
C6—C5—H5	120.3	H15A—C15—H15B	109.5
C4—C5—H5	120.3	O4—C15—H15C	109.5
C5—C6—C1	122.9 (2)	H15A—C15—H15C	109.5
C5—C6—H6	118.6	H15B—C15—H15C	109.5
C1—C6—H6	118.6	O5—C16—H16A	109.5
O3—C7—N1	120.7 (2)	O5—C16—H16B	109.5
O3—C7—C1	121.7 (2)	H16A—C16—H16B	109.5
N1—C7—C1	117.6 (2)	O5—C16—H16C	109.5
N2—C8—C9	121.6 (3)	H16A—C16—H16C	109.5
N2—C8—H8	119.2	H16B—C16—H16C	109.5

### Hydrogen-bond geometry (Å, °)

**Table:**

*D*—H···*A*	*D*—H	H···*A*	*D*···*A*	*D*—H···*A*
N1—H1A···O1	0.89 (1)	1.93 (3)	2.661 (3)	138 (4)
O1—H1···O3^i^	0.82	2.16	2.872 (3)	146
O2—H2···O3^ii^	0.82	1.90	2.706 (3)	166

Symmetry codes: (i) *x*, −*y*+1, *z*−1/2; (ii) *x*, −*y*+2, *z*−1/2.
